# Vaccination and Its Impact on Lung Involvement in COVID-19 Patients: A Retrospective Study in India

**DOI:** 10.7759/cureus.58904

**Published:** 2024-04-24

**Authors:** Suhasini Balasubramaniam, Priyadarsini Bose, Pravin Kumar Raviganesh, Pravin Pandian, Balaji Selvaraj, Rajasekaran Sivaprakasam, Sangeetha Balaji, Abhilekshmi AM, Priyadharshini Sivakumar, Swaminathan Ramasubramanian

**Affiliations:** 1 Radiodiagnosis, Government Stanley Medical College and Hospital, Chennai, IND; 2 Internal Medicine, Government Medical College, Omandurar Government Estate, Chennai, IND; 3 Pharmacology, Government Medical College, Omandurar Government Estate, Chennai, IND; 4 Radiodiagnosis, Government Medical College, Omandurar Government Estate, Chennai, IND

**Keywords:** vaccine hesitancy, india, covaxin, covishield, lung involvement, vaccination, sars-cov-2, covid-19

## Abstract

Background

COVID-19, caused by SARS-CoV-2, led to a global pandemic necessitating urgent vaccine development and deployment. By the end of 2020, several vaccines had reached their clinical trial endpoints. India, leveraging its pharmaceutical prowess, developed two primary vaccines: CoviShield® and Covaxin®. Despite the availability of these vaccines, vaccine hesitancy became a notable challenge. This study aimed to assess the correlation between vaccination status and lung involvement in COVID-19 patients, aiming to fortify trust in vaccines and enhance vaccine uptake in India.

Methods

This retrospective cross-sectional study analyzed data from 272 patients treated at a designated COVID-19 Care Center in Chennai, India, from May to July 2021. Patients were divided into vaccinated and unvaccinated groups, with vaccinated individuals further categorized based on the type and dose of vaccine received (CoviShield® or Covaxin®). Lung involvement was assessed through CT chest scans, and statistical analyses were performed to compare the severity of lung involvement across different groups.

Results

The vaccinated group demonstrated significantly lower mean lung involvement (28%) compared to the unvaccinated group (34.8%). Within vaccinated individuals, no significant differences were observed between different vaccine types and doses, suggesting a generalized protective effect of COVID-19 vaccination against severe lung involvement.

Conclusion

Vaccination against COVID-19 significantly reduces the severity of lung involvement among patients, irrespective of the vaccine brand or dose. This study reinforces the importance of vaccination in mitigating the impact of COVID-19 and supports ongoing vaccination efforts.

## Introduction

In late 2019, the world was introduced to an unforeseen challenge: the outbreak of the novel coronavirus, officially termed severe acute respiratory syndrome coronavirus 2 (SARS-CoV-2), originating in Wuhan, China. Rapidly, this virus metastasized beyond borders, taking a global form and escalating into the coronavirus disease 2019 (COVID-19) pandemic [1]. The severity and scale of this crisis were of such magnitude that it mandated an urgent response from the global scientific community. The race for a vaccine became a global endeavour, marked by unprecedented collaboration and resource allocation. By the close of 2020, multiple vaccines with varied underlying technologies had entered the final stages of their clinical trials, offering a beacon of hope to a world paralyzed by the pandemic [2].

India, with its vast pharmaceutical and biotechnological capabilities, emerged as a significant player in this global vaccine initiative [3]. Two vaccines, CoviShield® and Covaxin®, received emergency approval from the Indian government and played pivotal roles in the country's vaccination strategy. CoviShield®, developed by the Serum Institute of India® in collaboration with AstraZeneca® and the University of Oxford, utilizes the viral vector platform based on the ChAdOx1 nCoV-19 vaccine. It employs a harmless virus to deliver a segment of the SARS-CoV-2 genetic material, which encodes the spike protein, triggering an immune response to confer protection against the virus. Covaxin®, developed by Bharat Biotech® in partnership with the Indian Council of Medical Research (ICMR), is an inactivated virus vaccine. This involves using a deactivated form of the virus to stimulate an immune response without causing illness [4,5]. Vaccination has demonstrated efficacy in augmenting the immune response and diminishing the probability of subsequent infections in individuals with prior exposure. Nonetheless, the specific vaccination type and the individual's health status exert considerable influence on immune reactions [6].

As India commenced its phased vaccine rollout, an unanticipated challenge surfaced: vaccine hesitancy. Despite the staggering toll of the pandemic, many among the general public exhibited reluctance or outright refusal to get vaccinated. Preliminary surveys and anecdotal evidence suggested a myriad of reasons, ranging from concerns about side effects to mistrust in the speed of vaccine development [7-9].

Given this backdrop, our study seeks to provide a quantitative assessment of the benefits of vaccination against COVID-19, irrespective of the vaccine brand. Furthermore, we aim to evaluate potential disparities among vaccine brands concerning their association with lung involvement. By highlighting the efficacy and potential long-term advantages of getting vaccinated, we aim to dispel prevailing myths and hesitations.

## Materials and methods

Study setting and design

This retrospective cross-sectional analysis was conducted in a hospital setting, emphasizing the examination of CT chest scans. The Institutional Ethics Committee granted its consent under IEC No. 105/IEC/GOMC/2023. The study was in strict compliance with the ethical directives of the institutional research board and aligned with the ethical principles stated in the 1964 Helsinki Declaration and its later modifications or comparable ethical norms. The research was conducted by the Department of Radiodiagnosis at the Government Medical College located in Omandurar Government Estate, Chennai, which is associated with The Tamil Nadu Dr. M.G.R. Medical University and was recognized as an official COVID-19 Care Center during the initial stages of the pandemic.

Study period

The investigation spanned from May 2021 through July 2021, offering a defined period for an in-depth data review.

Participants

Subjects enrolled in the study were categorized based on their immunization status into two distinct cohorts: the vaccinated group and the unvaccinated group. Within the vaccinated group, individuals were further stratified into subgroups corresponding to their specific vaccination regimen, namely: recipients of the first dose of CoviShield® (CS1), recipients of the second dose of CoviShield® (CS2), recipients of the first dose of Covaxin® (CV1), and recipients of the second dose of Covaxin® (CV2).

Inclusion criteria

To be considered for this study, individuals had to be 18 years or older at the time of hospital admission. Additionally, they needed to present with clinical signs indicative of COVID-19, such as fever, cough, breathlessness, fatigue, muscle pain, headache, a loss of taste or smell, throat soreness, nasal congestion or discharge, nausea or vomiting, and diarrhea [10]. A key inclusion requirement was a positive confirmation of COVID-19 via reverse transcription-polymerase chain reaction (RT-PCR).

Exclusion criteria

Subjects under 18, those without COVID-19 symptoms, or without a positive RT-PCR confirmation for COVID-19 were excluded from the study [10].

Sample size

From the eligible pool, 272 patients were chosen through convenience sampling to meet the defined inclusion criteria.

CT chest imaging protocol

Chest CT scans without contrast were conducted with patients lying on their backs using a 16-slice multidetector CT scanner (Aquilion Lightning model TSX-035A, Toshiba America Medical Systems, Tustin). During scanning, patients were instructed to hold their breath. The Vitrea software (version 6.5.99; Vital Images, Inc., Otawara, Japan) was used for image evaluation, with a window width of 1,000-2,000 Hounsfield units (HU) and a level setting of -700 to -500 HU. Scans encompassed the full lung area from top to bottom. Initial analysis was performed by two radiologists with a combined experience of 15 years, with further review involving residents and interns in a double-blind manner. Any interpretative disputes were settled by a leading radiologist with 20 years of experience.

Quantification of pulmonary involvement

Lung involvement (LI) was quantified across 20 sections, with scores assigned based on the extent of opacification: two points for more than half involvement and one for less. The maximum achievable score was 40, where each point corresponded to 2.5% of the lung tissue affected [11].

Data management

Data from CT scans and patient files were meticulously recorded into a Microsoft Excel spreadsheet (Microsoft® Corp., Redmond, Washington, DC), ensuring strict confidentiality and privacy standards.

Statistical analysis

Statistical analysis was conducted using Python 3.9 (Python Software Foundation, Wilmington, DE) with the assistance of several libraries. *Pandas* was utilized for data organization and manipulation, *numpy* for numerical analysis, *scipy* for conducting statistical tests, and *matplotlib* and *seaborn* for graphical presentations, including *statsmodels* for advanced statistical models such as ANOVA and interaction effects. Initially, the dataset was prepared and cleaned using *pandas*, and descriptive statistics were used to provide an overview of LI across the study population. Differences in LI between vaccinated and unvaccinated cohorts were highlighted by calculating means, standard deviations, and other statistics across various demographic and vaccination categories. The statistical analysis included an ANOVA to evaluate the significance of differences in average LI between different age groups and between sexes, aiming to determine if demographic factors influenced the severity of LI. Additionally, interaction effects were analyzed using ANOVA to assess if there were significant interactions between sex and vaccination status on LI, incorporating interaction terms into the model to explore combined effects. Further detailed subgroup analyses were performed on the vaccinated cohort, segmented by vaccine type (CoviShield® vs. Covaxin®) and dosage. Tukey's honest significant difference (HSD) test was used to evaluate mean differences among all vaccine subgroup pairings to identify statistically significant differences in LI among different vaccine types and dosages. Graphical presentations were created to visually represent the distribution of LI across different vaccination statuses and demographic groups.

Ethical considerations

This study maintained strict adherence to the ethical standards of our institution, ensuring the confidentiality and anonymity of patient information. To maintain anonymity, patient identifiers were removed using Picture Archiving and Communications System (PACS) software (GE Healthcare, Chicago, IL), with unique random numbers assigned to each image and clinical record for linkage while preserving privacy. Data were stored securely in the Department of Radiodiagnosis’s computer system within a password-secured folder. All human participant activities were conducted respecting the ethical guidelines of both the institutional and/or national research committee.

## Results

Overall findings

We analyzed the demographic and clinical characteristics of 272 individuals, categorized by age, sex, and vaccination status. The participants ranged in age from 23 to 92 years, with an average age of approximately 54.3 years. The cohort included 164 males and 108 females. The cohort was segmented into vaccinated and not vaccinated groups. The vaccinated group comprised individuals who had received any COVID-19 vaccine dose, while the not-vaccinated group had not received any COVID-19 vaccine. Regarding vaccination status, our cohort was divided into those who received the COVID-19 vaccine and those who did not. Among the vaccinated individuals, 39 had received the first dose of CoviShield®, 12 the second dose of CoviShield®, 26 the first dose of Covaxin®, and 18 the second dose of Covaxin®. The majority of the cohort, 177 individuals, had not received any COVID-19 vaccine. The overall LI mean was observed to be 32.4%, with a standard deviation of 21.8%, highlighting a substantial variation in LI among the population. This has been summarized in Tables 1-2.

**Table 1:** Demographic Characteristics of Study Participants

Demographic Feature	Metric	Value
Overall	Count	272
	Mean Age	54.3
	Age Range	23-92
Sex	Male	164
	Female	108
Age Group	Less than 40	52
	40-60	125
	Greater 60	95
Vaccine Status	Not Vaccinated	177
	CoviShield® 1st Dose	39
	CoviShield® 2nd Dose	12
	Covaxin® 1st Dose	26
	Covaxin® 2nd Dose	18

**Table 2:** Overall Lung Involvement Statistics LI - Lung Involvement

Metric	Value
Count	272
Mean - LI (%)	32.4
Standard Deviation - LI (%)	21.8
Minimum - LI (%)	2.5
25th Percentile - LI (%)	15
Median - LI (%)	30
75th Percentile - LI (%)	47.5
Maximum - LI (%)	95

Vaccine status analysis

The vaccinated group (N=95) showed a lower mean LI (28%) compared to the not-vaccinated group (N=177), which had a mean LI of 34.8%. This difference suggests a potential protective effect of vaccination against severe LI. Despite the vaccinated group's wide range of LI (from 2.5% to 95%), the median value (20%) was significantly lower than that of the not vaccinated group (35%). This has been summarized in Table 3.

**Table 3:** Descriptive Statistics by Vaccination Status LI - Lung Involvement

Vaccination Status	Count	Mean - LI (%)	Standard Deviation - LI (%)	Minimum - LI (%)	25th Percentile - LI (%)	Median - LI (%)	75th Percentile - LI (%)	Maximum - LI (%)
Not Vaccinated	177	34.8	22.1	2.5	15	35	50	90
Vaccinated	95	28	20.7	2.5	14	20	41.25	95

Grouped statistics by vaccine status

**Figure 1:**
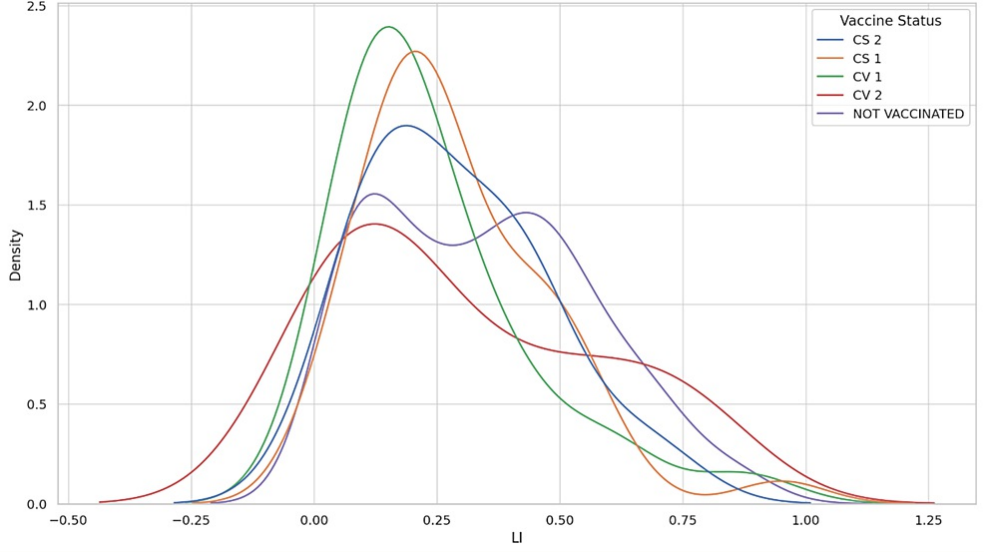
Density Plot of Lung Involvement by Vaccine Status LI - Lung Involvement, CS 1 - CoviShield® 1st dose, CS 2 - CoviShield® 2nd dose, CV 1 - Covaxin® 1st dose, CV 2 - Covaxin® 2nd dose This graph displays the probability density of lung involvement for different vaccination statuses. The peaks of the curves represent the most common LI values for each group. The presence of multiple peaks in some of the curves indicates a multimodal distribution, suggesting that there are several common LI values within that group.

**Figure 2:**
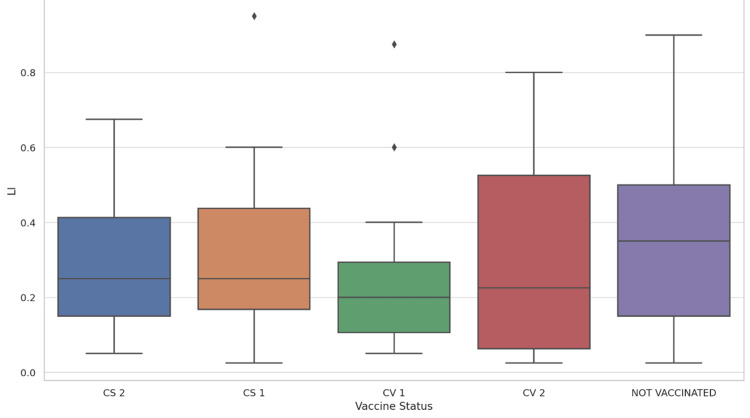
Box Plot of Lung Involvement vs Vaccine Status LI - Lung Involvement, CS 1 - CoviShield® 1st dose, CS 2 - CoviShield® 2nd dose, CV 1 - Covaxin® 1st dose, CV 2 - Covaxin® 2nd dose This box plot provides a five-number summary (minimum, first quartile, median, third quartile, and maximum) of the LI values for each vaccine status group, along with potential outliers represented by diamonds.

Detailed statistics for each vaccine subgroup revealed similar mean LI for CoviShield® 1st dose (28.7%) and CoviShield® 2nd dose (28.7%), and a slightly lower mean for Covaxin® 1st dose (24.5%). Covaxin® 2nd dose recipients showed a higher mean LI (31%), which might reflect variability due to the smaller sample size or other confounding factors. The standard deviations across these groups indicated a broad range of LI outcomes within each vaccination status category. This has been summarized in Table 4, Figure 1, and Figure 2.

**Table 4:** Statistics by Specific Vaccine Status LI - Lung Involvement

Vaccine Status	Count	Mean - LI (%)	Standard Deviation - LI (%)
CoviShield® 1st dose (CS 1)	39	28.7	18.8
CoviShield® 2nd dose (CS 2)	12	28.7	18.3
Covaxin® 1st dose (CV 1)	26	24.5	19.9
Covaxin® 2nd dose (CV 2)	18	31.0	27.4

Statistical analysis

**Figure 3:**
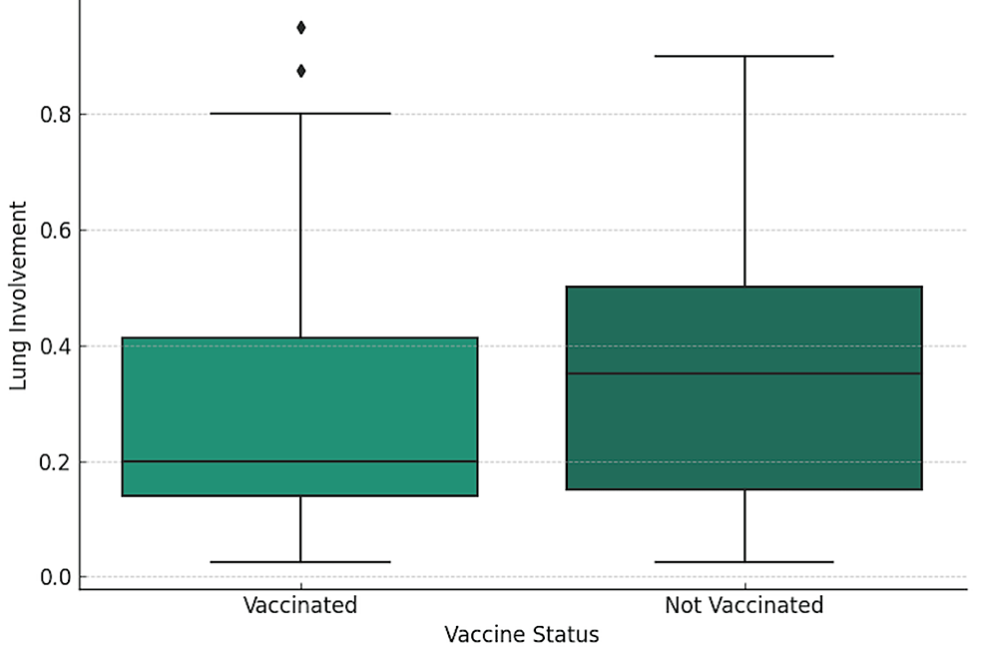
Lung Involvement by Vaccine Status This graph compares the distribution of lung involvement between two categories: vaccinated and not vaccinated.

ANOVA was conducted to compare the mean LI between the vaccinated and not vaccinated groups. It yielded a significant F-statistic of 6.079, with a p-value of 0.0143, indicating a statistically significant difference in LI between the two groups (Figure 3). This result supports the hypothesis that vaccination status is associated with the extent of LI, with vaccinated individuals showing lower mean LI than those not vaccinated.

Further analysis was conducted to dissect the vaccinated group into subgroups based on the type of vaccine received and the dose number. No significant mean differences were found between the different vaccine subgroups (CoviShield® 1st dose vs. CoviShield® 2nd dose, CoviShield® vs. Covaxin®, etc.), as indicated by the adjusted p-values, which were all well above the 0.05 significance threshold. This lack of significant difference within vaccinated subgroups suggests that the protective effect against severe LI might not be strongly dependent on the type of vaccine or the dose number. This has been summarized in Table 5.

**Table 5:** Tukey's Honestly Significant Difference Test CS 1 - CoviShield® 1st Dose, CS 2 - CoviShield® 2nd Dose, CV 1 - Covaxin® 1st Dose, CV 2 - Covaxin® 2nd Dose, CI - Confidence Interval

Vaccine Groups Compared	Mean Difference	Adjusted P-value	Lower Bound CI	Upper Bound CI	Significant Difference
CS 1 vs. CS 2	-0.0002	1.0000	-0.1971	0.1967	No
CS 1 vs. CV 1	-0.0421	0.9401	-0.1931	0.1089	No
CS 1 vs. CV 2	0.0224	0.9963	-0.1475	0.1924	No
CS 2 vs. CV 1	-0.0419	0.9815	-0.2500	0.1663	No
CS 2 vs. CV 2	0.0226	0.9987	-0.1996	0.2449	No
CV 1 vs. CV 2	0.0645	0.8689	-0.1184	0.2474	No

LI analysis by age and sex

Our analysis indicated that age and sex did not significantly influence the extent of LI in individuals post-vaccination. While age groups were segmented into "under 40", "40 to 60", and "over 60", ANOVA results showed no significant differences in LI across these categories (F-statistic = 0.87, p = 0.422). This suggests that the protective effect of the vaccines is consistent across different age demographics within the context of our study cohort. Similarly, the interaction effect between sex and vaccine status was explored to determine if gender differences might affect vaccine efficacy concerning LI. However, the statistical analysis did not reveal any significant interaction (p = 0.213), implying that the vaccine’s effectiveness against LI is uniform across sexes within this dataset.

Subgroup analysis by vaccine status

Descriptive statistics highlighted differences in LI across vaccinated and not-vaccinated groups, with the vaccinated group showing a lower mean LI. This suggests a potential protective effect of the COVID-19 vaccines. However, the lack of significant differences in LI between different vaccines and doses within the vaccinated group, as indicated by subgroup analysis, supports the notion that the primary factor is vaccination itself rather than the type of vaccine or the number of doses administered.

## Discussion

The findings from this retrospective analysis underscore the pivotal role vaccinations may play in modulating disease outcomes, particularly with regard to LI. On analyzing the data from the 272 patients, an overarching trend emerges: the vaccinated group demonstrated significantly lower mean LI (28%) compared to the unvaccinated group (34.8%). This disparity lends weight to the notion that vaccination may confer a protective effect against the more severe manifestations of the disease.

Studies from various regions provide comprehensive insights into the efficacy of COVID-19 vaccinations. These studies collectively highlight the substantial effectiveness of vaccination in reducing infection rates, severity of illness, and hospital stays [5,12,13]. Vaccination, whether partial or complete, demonstrates notable efficacy, with partial vaccination offering 52% protection and full vaccination providing 83% protection against SARS-CoV-2 infection [5,12]. Moreover, complete vaccination substantially decreases the likelihood of severe cases, as evidenced by reduced hospitalization rates and diminished risks of severe adverse events post-vaccination [5]. Notably, previous COVID-19 infection enhances vaccine effectiveness, further bolstering immunity, even against severe infections [5]. Research comparing different vaccine modalities, including mRNA and adenovirus vector vaccines, suggests consistent effectiveness in reducing infections, hospitalizations, and mortality rates [14]. These findings underscore the pivotal role of vaccination in mitigating the impact of the COVID-19 pandemic and underline the need for continued research to optimize vaccine strategies and enhance global vaccine access [13,14]. Discussions on vaccine safety and immune responses highlight the importance of proper vaccination alignment with patients' cases to ensure optimal outcomes [6,15]. Additionally, systematic reviews evaluate the efficacy and safety of COVID-19 vaccines, providing insights into various outcomes such as confirmed symptomatic COVID-19 and all-cause mortality while assessing the certainty of evidence using the grading of recommendations assessment, development and evaluation (GRADE) approach [15].

Despite observable differences in LI across vaccinated cohorts, statistical analyses, namely, the one-way ANOVA and Tukey's HSD test, did not pinpoint any significant disparities between the brands of vaccines. This suggests that, concerning LI, different vaccines might exhibit analogous efficacies.

CoviShield®, an adenovirus vector vaccine, demonstrates a varying efficacy range across studies, with reported figures ranging from 55.1% to 100%, depending on the interval between doses [12,13,16]. On the other hand, Covaxin®, an inactivated virus vaccine, exhibits consistent efficacy rates, with studies reporting figures around 77.8%-81% [6,12]. CoviShield®'s platform, based on the ChAdOx1nCov-19 vaccine, offers the advantage of easier storage and transportation compared to Covaxin® [6,17]. However, Covaxin® has shown a higher efficacy rate in certain contexts and may induce lower side effects, according to comparative reviews [6,18]. Real-world studies, such as those conducted in Mumbai, demonstrate Covishield®'s higher effectiveness in preventing COVID-19 infections, likely contributing to its wider uptake compared to Covaxin® [5,6].

Being retrospective, there is an inherent risk of biases. The data reveal a correlation, but causality remains elusive and would necessitate more rigorously controlled investigations. Moreover, the specific vaccines - CoviShield® 1st dose, CoviShield® 2nd dose, Covaxin® 1st dose, Covaxin® 2nd dose - do not display significant variances in protection, but the smaller cohorts in subgroups might be a statistical constraint. Hence, larger, more balanced studies or meta-analyses could offer a clearer lens on this. The data lack information regarding the time elapsed since the dose was administered, which represents a potential confounding factor. Additionally, potential confounders such as underlying comorbid conditions, and the time elapsed between vaccination and subsequent infection are conspicuously absent but could be pivotal in understanding the relationship between vaccination and LI.

In light of these findings, it is paramount for health authorities to persistently champion vaccination as a cornerstone in mitigating severe disease trajectories. However, a granular exploration into individual vaccines, their timings, and specific outcomes is still warranted. Future research endeavors should emphasize balanced cohort compositions, contemplate all conceivable confounding factors, and might benefit from a prospective design to circumvent biases characteristic of retrospective studies.

## Conclusions

The findings of this retrospective study underscore the protective effect of COVID-19 vaccination on LI among patients in India, with vaccinated individuals exhibiting significantly lower LI than their unvaccinated counterparts. Despite the analysis encompassing various vaccine types and dosages, no substantial differences were observed among the specific vaccine subgroups. This suggests that the protective benefits against severe LI are broadly applicable across different COVID-19 vaccines. This study contributes to the growing body of evidence supporting the efficacy of vaccination in reducing the severity of COVID-19 outcomes. It highlights the importance of widespread vaccine uptake as a critical measure in the public health response to the pandemic.
